# Heads-up Digitally Assisted Surgical Viewing with Intraoperative Optical Coherence Tomography for Myopic Schisis Repair

**DOI:** 10.18502/jovr.v16i1.8259

**Published:** 2021-01-20

**Authors:** Renato Menezes Palácios, Kim Vieira Kayat, Michel Eid Farah, François Devin

**Affiliations:** ^1^Department of Ophthalmology-Retina, Federal University of São Paulo, São Paulo, Brazil; ^2^Department of Ophthalmology-Retina, Centre Monticelli Paradis d'Ophtalmologie, Marseille, France

**Keywords:** Heads-up surgery, 3-D, Intraoperative Optical Coherence Tomography, Myopic Macular Schisis, Foveal-sparing Internal Limiting Membrane Peeling

## Abstract

**Purpose:**

To describe the surgical approach with a screen-based heads-up, three-dimensional (3-D) digital viewing with intraoperative optical coherence tomography (I-OCT) for the successful repair of a myopic macular schisis (MMS) case.

**Case Report:**

A 62-year-old woman with vision loss in the left eye was scheduled for pars plana vitrectomy (PPV) and MMS repair. Surgery was performed using the NGENUITYⓇ system for surgical viewing, and foveal-sparing internal limiting membrane (fs-ILM) peeling was performed without gas tamponade, after confirming the absence of iatrogenic macular hole with I-OCT. There were no intraoperative or postoperative complications. Visual acuity improved to 20/40 and the subfoveal macular thickness improved from 706 µm (preoperative) to 221 µm after seven months of follow-up.

**Conclusion:**

Heads-up digitally assisted viewing technology with I-OCT may be useful or preferred for patients requiring vitreoretinal surgery in the setting of MMS.

##  INTRODUCTION

**Figure 1:**
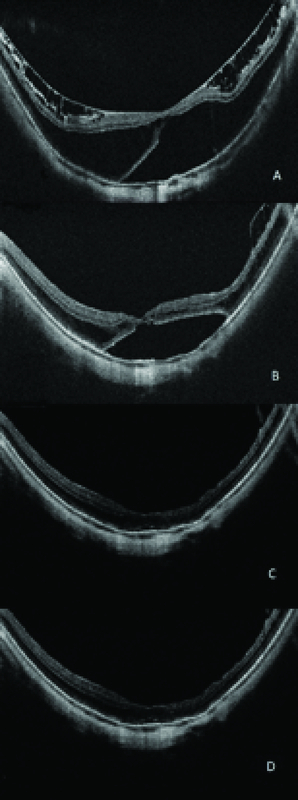
Intraoperative snapshot showing fs-ILM peeling using 3-D surgical viewing.

Myopic macular schisis (MMS) is a pathology that is typically seen in high myopic patients, which is distinguished by progressive secession of the neurosensory retinal layers. Many articles have shown that pars plana vitrectomy (PPV), with or without internal limiting membrane (ILM) peeling and gas tamponade, is a successful treatment for this condition.^[1,2,3,4]^ Here, we describe an MMS case treated with PPV and foveal-sparing internal limiting membrane (fs-ILM) peeling [Figure 1], without gas tamponade, using intraoperative optical coherence tomography (I-OCT) and digitally-assisted vitreoretinal three-dimensional (3-D) viewing.

**Figure 2:**
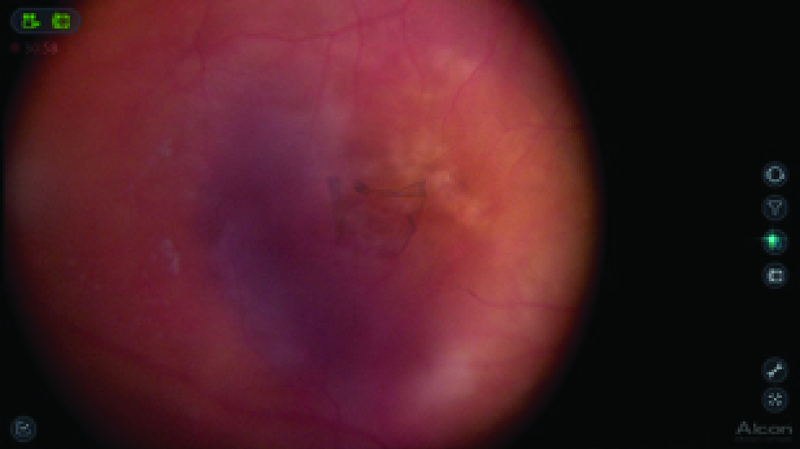
Intraoperative snapshot showing fs-ILM peeling using 3-D surgical viewing and I-OCT. The I-OCT shows no evidence of any iatrogenic complication.

**Figure 3:**
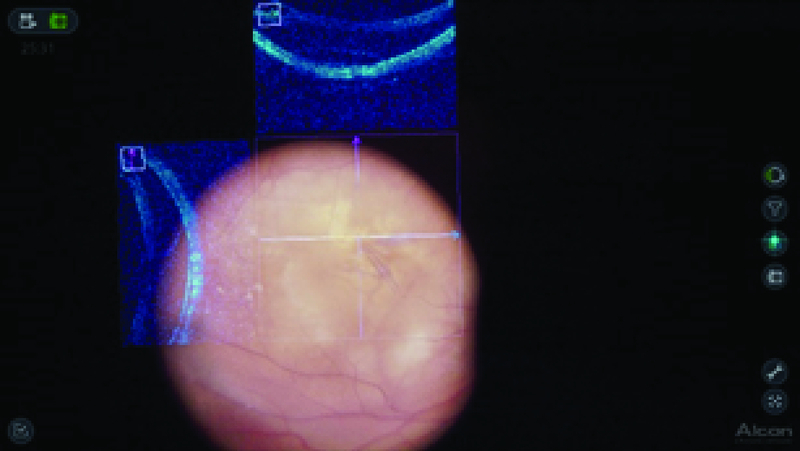
(A) Preoperative appearance, (B) partial resolution of the macular schisis one month postoperatively, and complete resolution of the schisis (C) four and (D) seven months after the surgery. Visual acuity improved from 20/200 to 20/40 and subfoveal macular thickness improved from 706 to 221 µm after seven months of follow-up.

##  CASE REPORT

A 62-year-old woman with MMS in the left eye of few month duration underwent a complete ophthalmologic examination that included best-corrected visual acuity (BCVA), slit-lamp biomicroscopy, fundus examination, and applanation tonometry. Spectral domain optic coherence tomography (SD-OCT) images were obtained with Cirrus HD-OCT (Carl Zeiss AG, Oberkochen, Germany) at baseline and at all follow-up visits (one, four, and seven months). Subfoveal macular thickness was 706 micrometers (µm), vision was 20/200, and PPV was scheduled. The patient had a history of phacoemulsification in the left eye.

The anesthetists performed sedation and a retrobulbar block. The NGENUITYⓇ digitally assisted vitreoretinal surgery system (Alcon, Inc., Fort Worth, TX) was connected to replace the oculars of the microscope. The 3-D high definition real-time video was displayed on the NGENUITYⓇ4K 3-D flat-panel placed at 1.3 m from the surgeon. To be able to see in 3-D, the surgeon wore polarized glasses. Traditional vitreoretinal techniques, with the Constellation Vision System (Alcon, Inc, Fort Worth, TX), were performed without obstacles, including core vitrectomy, posterior hyaloid detachment, and peripheral vitrectomy. Brilliant blue G (DORC, Zuidland, the Netherlands) was used to stain the ILM and the surgeon performed fs-ILM peeling using disposable 25-gauge end-grasping forceps under I-OCT [Figure 2]. The I-OCT also proved that there were no iatrogenic lesions [Video 1], so it was decided not to perform gas tamponade. The subfoveal macular thickness improved from 706 µm (preoperative), 540 µm (after one month), 214 µm (after four months) to 221 µm (after seven months) [Figure 3] and the visual acuity improved to 20/40 after seven months of follow-up.

##  DISCUSSION

In the current case, a 62-year-old woman was scheduled for PPV and MMS repair in the left eye, using the 3-D system. Foveal sparing ILM peeling was performed, without gas tamponade, after confirming the absence of iatrogenic macular hole with I-OCT.

MMS has already been described by many authors, showing a wide variety of therapeutic interventions. In most reported cases in which PPV was performed, ILM peeling was advised to completely remove residual traction on the retina, enabling the inner surface to adjust to the mold of the posterior staphyloma.^[2]^ It is still a surgical challenge to measure accurately the dimensions of ILM sparing intraoperatively.

With the development of 3-D system^[5]^ with I-OCT, real-time visualization of vitreoretinal interface, definition of the various plans of epiretinal membranes (ERM) and macular holes (MH), and visualization of ILM undulation after successful peeling can help in unequalled exactitude in an otherwise assumptive surgery.^[6]^ Visualization of resolution of traction following vitrectomy and ERM removal can also help determine the surgical termination. Addition of such an advance would further improve management of MMS to very small precision.^[3]^ In the current case, similar to Kumar et al,^[3]^ fs-ILM peeling was performed under direct I-OCT visualization of the requisite area of sparing to prevent intraoperative deroofing of the cysts and MH formation.

Gas tamponade has been used in the treatment of MMS, provoking retinal repositioning by pushing the retina back. However, it remains unknown whether gas tamponade is necessary and its efficacy has not been established. Kim et al^[4]^ showed resolution of MMS in six of the eight eyes (75.0%) after PPV and ILM peeling without gas tamponade. On the other hand, Figueroa et al^[2]^ achieved resolution of MMS in 93% of 30 patients after PPV with ILM peeling and gas tamponade.

Our case demonstrates that heads-up digitally assisted viewing with I-OCT was suitable and effective to manage these challenging retinal disorders.

##  Financial Support and Sponsorship

None.

##  Conflicts of Interest

There are no conflicts of interest.
